# Complete Cure of Inoperable Stage IV Locally Advanced Hypopharyngeal Squamous Cell Carcinoma by an Innovative Combination Cancer Immunotherapy Consisting of Radiation, Immune Checkpoint Inhibitors, and Dendritic Cell Vaccine

**DOI:** 10.7759/cureus.69429

**Published:** 2024-09-14

**Authors:** Takanori Iwasaki, Yuichiro Yazaki, Tomohiro Myojo, Takashi Masuko, Takashi Nishimura

**Affiliations:** 1 Cancer Therapy, Precision Clinic Group, Tokyo, JPN; 2 Pharmacy, Kindai University, Higashiosaka, JPN; 3 Tumor and Gene Regulation, Oncology Innovation Center, Fujita Health University, Nagoya, JPN; 4 Cancer Immunotherapy, Precision Clinic Group, Tokyo, JPN

**Keywords:** cancer peptide vaccine, combination cancer immunotherapy, cytotoxic t lymphocytes, dendritic cell vaccine, hypopharyngeal cancer, immune-checkpoint inhibitors, radioimmunotherapy, type1-helper t cells

## Abstract

We report the case of a 68-year-old man with locally advanced (LA) head and neck cancer (HNC) (LA-HNC). The patient was diagnosed with inoperable stage IVA hypopharyngeal squamous cell carcinoma with 2 cm primary and three lymph node metastatic cancers. The patient was treated with an innovative combination cancer immunotherapy (iCCI) consisting of radiotherapy, immune checkpoint inhibitors, and helper/killer-hybrid epitope long peptides (H/K-HELP)-pulsed dendritic cell vaccine. These three treatments constituting iCCI are known to show an immunomodulating effect on tumor-draining lymph nodes (TDLNs) and improve antitumor immunity in tumor microenvironments (TMEs) to reduce tumor growth. Surprisingly, the patient treated with iCCI showed a complete cure for all the cancers including primary and lymph node-metastatic cancers without standard chemotherapy. The patient is still cancer-free for almost two years. Although the destruction mechanism of cancer is not determined, we speculate this iCCI might improve the patient’s antitumor immune capability around tumor sites including TDLNs and TME. Our developed iCCI will become a promising strategy to overcome inoperable cancers in the future.

## Introduction

Head and neck cancers (HNC) are one of the most common cancers worldwide and a vast majority of HNCs are head and neck squamous cell carcinomas (HNSCC), which have poor prognosis [1,2]. Generally, locally advanced (LA)-HNSCC has been primarily treated with radiotherapy combined with or without chemotherapy, but the discovery of immune checkpoint inhibitors (ICIs) changed the paradigm of cancer treatment and combination therapy with radiation, and ICIs are becoming the standard part of treatment of patients with intractable cancers including LA-HNSCC and recurrent/metastatic HNC [3]. However, only subsets of cancer patients benefit from the combination therapy with radiation and ICIs, but many fail to develop durable responses [4]. Therefore, developing a more powerful combination cancer immunotherapy that can completely cure cancers is necessary.

The accumulating evidence shows that tumor-draining lymph nodes (TDLNs) rather than tumor microenvironment (TME) play key roles in antitumor T cell responses that can be induced following various cancer immunotherapies, such as radiotherapy, ICIs therapy, and cancer vaccine therapy [5-8]. We initially found the crucial role of tumor-specific Th1 cells but not Th2 cells in tumor immunology [9]. We demonstrated that the initial activation of interferon gamma-producing (IFN-γ-producing) CD4^+^ Th1 cells in TDLNs is crucial for overcoming strong immunosuppression and for inducing a complete rejection of solid tumor by CD8^+ ^CTL [5,7,8,10]. The essential role of IFN-γ-producing Th1 cells was recently confirmed even in a neoantigen-positive cancer therapy model [11]. Based on these findings, we hypothesized that it might be possible to effectively modulate antitumor immunity in TDLNs and introduce Th1-dominant immunity, which is essential for inducing a complete rejection of solid tumors by cytotoxic T lymphocytes (CTL) by creating a better-combined cancer therapy. Here, we developed an innovative combination cancer immunotherapy (iCCI) consisting of radiotherapy, ICIs, and dendritic cell (DC)-based cancer vaccine (DC_VAC_).

For the past decades, the main mechanism of tumor growth inhibition after radiotherapy was attributed to radiation-induced tumor DNA damage. However, in previous papers [5], we demonstrated that radiation-induced tumor reduction is because of radiation-induced CD8^+ ^tumor-specific CTL generated in TDLNs. Furthermore, we showed the therapeutic effect of radiotherapy was greatly augmented by the reprogramming of immunosuppressive TDLNs and TME by CD4^+ ^tumor-specific Th1 cells. Therefore, radiotherapy might be a good candidate for constructing iCCI if we could incorporate other cancer therapies, which can introduce Th1-dependent antitumor immune responses in TDLNs. Radiotherapy can modify TME from immunosuppressive to immunostimulatory states [4]. Namely, radiation-induced immunogenic cell death (ICD) activates antigen-presenting cells, which facilitate the induction of Th1 and CTL specific to neoantigen and common cancer-rejection antigens such as WT1 and survivin. Therefore, it is a rational strategy to combine radiotherapy with immunotherapy.

Recently, it was reported that PD-1/PD-L1 interaction in TDLNs, but not TME, correlated with prognosis in melanoma patients [6]. Moreover, the specific targeting of PD-L1 in TDLNs induced an effective antitumor-immune response in multiple tumor-therapeutic models [6]. IFN-γ-producing CD4^+^T cells are required for the efficacy of ICI therapy [11]. It is also reported that both ipilimumab, a blockade of cytotoxic T-lymphocyte associated protein 4 (CTLA-4), and nivolumab, an inhibitor of PD-1, are involved in the activation of Th1-immunity [11,12]. Thus, ICI therapy is a good candidate for constituting iCCI to modulate Th1-dependent antitumor immunity in TDLNs.

Cancer treatment with helper/killer-hybrid epitope long peptides (H/K-HELP)-pulsed DC_VAC_ is selected as a third candidate to construct iCCI. Our developed H/K-HELP, which contains both class I- and class II-binding cancer peptides, has been shown to induce Th1-dependent antitumor immunity in TDLN essential for destroying solid tumors in a preclinical study [8]. Moreover, in clinical studies, we have shown that survivin-H/K-HELP could induce tumor-specific Th1 cells and CTL to induce a complete response in triple-negative breast cancer patients [13]. The advantage of using H/K-HELP-DC_VAC_ is that antigen-pulsed activated DCs readily migrate to the TDLN and CD4^+ ^and CD8^+ ^T cells respond to DCs presenting WT-1 and survivin, which are expressed in most cancers, resulting in effective cancer therapy. This is because H/K-HELP-pulsed DC_VAC_ is expected to be a good cancer treatment for inducing stronger Th1-dependent antitumor immunity against WT-1 and survivin common cancer antigens in addition to neoantigens released by radiotherapy-induced ICD.

Here, we will highlight a successful outcome of a stage IV inoperable hypopharyngeal cancer patient treated with iCCI consisting of radiotherapy, low-dose ICIs, and H/K-HELP-DC_VAC_.

## Case presentation

**Figure 1:**
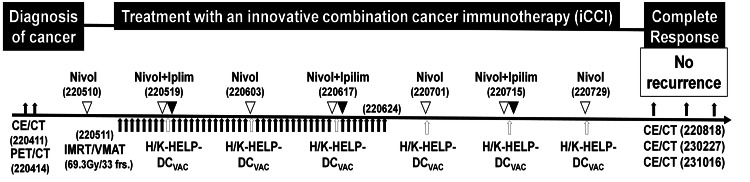
The therapeutic protocol for an innovative combination cancer immunotherapy (iCCI) After diagnosis with the endoscopy (pharynx to esophagus region), CECT, and PET-CT, the patient was treated with iCCI consisting of radiotherapy, ICIs and DC vaccine pulsed with helper/killer hybrid epitope long peptide (H/K-HELP-DC_VAC_). A day after nivolumab infusion (220511 white arrowheads), the patient was treated with intensity-modulated radiotherapy/volumetric modulated arc therapy (IMRT/VMAT: 69.3Gy in 33 fractions, black arrows). In addition to radiotherapy, the patient was treated with H/K-HELP-DC_VAC_ (white arrows) combined with low doses of nivolumab (40 mg fixed dose) alone or both nivolumab (40 mg fixed dose) and ipilimumab (20 mg fixed dose) (black arrowheads). The combined treatment was administered six times at two-week intervals. Mature DCs, which were pulsed with both WT-1-H/K-HELP and survivin-H/K-HELP, were used as H/K-HELP-DC_VAC_.

**Figure 2:**
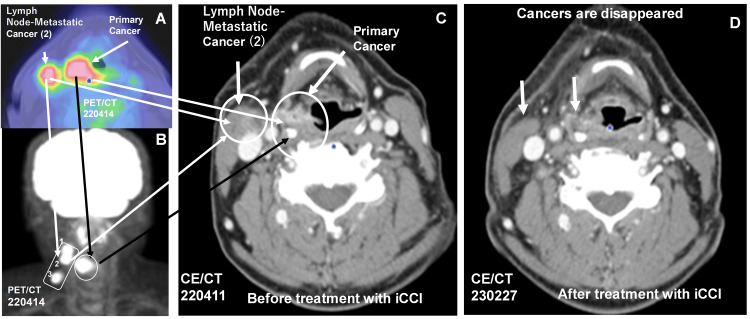
Complete response of locally advanced hypopharyngeal squamous cell carcinoma by treatment with an innovative combination cancer immunotherapy (iCCI) comprising radiotherapy, immune checkpoint inhibitors (ICIs), and dendritic cell (DC) vaccine pulsed with helper/killer hybrid epitope long peptide The patient was diagnosed as inoperable stage IVA hypopharyngeal squamous cell carcinoma with 2 cm primary and three lymph node (LN) metastatic cancers by PET-CT (A, cancer lesions were indicated by arrows; B, primary cancer is indicated by black arrow with circle and three LN lesions (1, 2, 3) are indicated by a white arrow with rectangle] and CECT; C, primary and LN metastatic cancer (2) lesions were indicated by white arrows with circles). The outcome of iCCI treatment against advanced HNSCC was diagnosed by CECT on August 18, 2022 (D), and it was demonstrated that all cancers, including the primary and three LN metastatic cancers on the right side of the neck, completely disappeared (D, the disappearance of cancer 2 were indicated by arrows and the disappearance of other two cancers (1 and 3) are indicated in Figure 3) by treatment with iCCI. No recurrence has been confirmed by CECT on February 27, 2023, and October 16, 2023.

**Figure 3:**
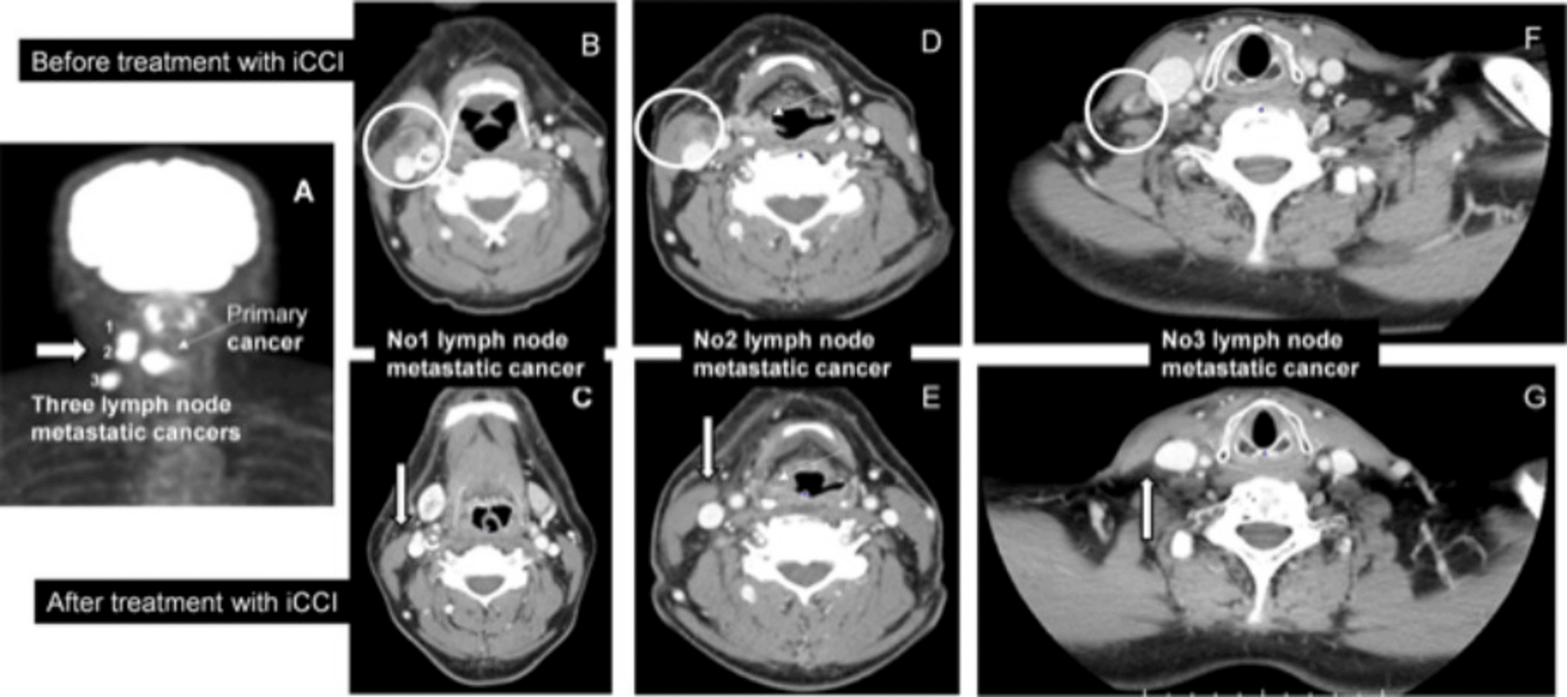
A complete disappearance of the three lymph node cancers on the right side of the neck by treatment with an innovative combination cancer immunotherapy (iCCI) The patient suffered from primary hypopharyngeal squamous cell carcinoma (A, D; thin arrow) with three LN-metastatic cancers on the right side of the neck (A, white arrow, and numbers 1-3) and was treated with iCCI. Before treatment with iCCI, LN-metastatic cancers (B, No. 1 cancer; D, No. 2 cancer; F, No. 3 cancer) were detected by CECT (indicated by circles). Here, we showed a complete disappearance of three LN-metastatic cancers (C: No. 1, E: No. 2, G: No. 3 cancer) after iCCI treatment. Lesions of disappeared LN-metastatic cancers are indicated by white arrows (C, E, and G). No recurrence of metastatic cancers occurred for over 18 months until now.

A new cancer treatment was designed and implemented in a patient according to a therapeutic protocol illustrated in Figure 1. A 68-year-old man feeling a physical disorder in the inner part of his neck visited a clinic on April 8, 2022. Although the patient had little history of smoking (only a few months when he was a college student), he had a long history of alcohol consumption, which is one of the risk factors for pharyngeal cancer. Although human papillomavirus (HPV) has been reported as one of the risk factors of HNCs, we did not test for HPV infection in the patient's hypopharyngeal cancer. Because it is well known that there is a causal relationship between HPV infection and the onset of oropharyngeal cancer, this is still unclear in the case of hypopharyngeal cancer. By the endoscopy and contrast-enhanced CT (CECT) on April 11 (Figure 2C) and positron emission tomography (PET) and CT (PET-CT) scan on April 14 (Figures 2A-2B), approximately 2 cm hypopharyngeal cancer with three LN metastatic cancers were found in cervical LNs on the right side of the neck (Figures 2A-2C and Figures 3A, 3B, 3D, 3F). The primary cancer was located at the right pyriform sinus with a slight involvement of the esophagus, but the oropharynx was intact. Pathological examination revealed that the tumor was well-differentiated squamous cell carcinoma; clinical diagnosis based on Union for the International Cancer Control (UICC) tumor, node, metastasis (TNM) classification in the eighth edition was T2aN2bM0 stage IVA.

On May 11, 2022, a day after an intravenous infusion of nivolumab (20 mg fixed dose), the patient was treated with IMRT/VMAT: 69.3 Gy in 33 fractions). The patient continued IMRT/VMAT (2.1 G/day) every day around the primary tumor and three LN metastatic cancers until June 24. From May 19, the patient received iCCI including IMRT, ICIs, and H/K-HELP-DCVAC, but excluding standard chemotherapy. Nivolumab (40 mg fixed dose) alone or both nivolumab (40 mg fixed dose) and ipilimumab (20 mg fixed dose) were infused to the patient every two or four weeks, respectively. Lower doses of ICIs were used to reduce adverse effects [14]. DC was prepared from the patient’s peripheral blood mononuclear cells as described previously [15]. DC pulsed with both WT-1-H/K-HELP(RMFPNAPYLGGGGGKRYFKLSHLQMHSRKHGGGGGCYTWNQMNL) and survivin-H/K-HELP(EHKKHSSGCAFLSVKKQFEGGGGGGFLKLDRERAKNKGGG) was used as H/K-HELP-DC vaccine (H/K-HELP-DCVAC). The human leukocyte antigens (HLA) haplotype of this patient was completely matched to the H/K-HELP of both WT1 and survivin. H/K-HELP-DCVAC was intradermally injected around the tumor site every two weeks. After completion of IMRT/VMAT, the patient continued H/K-HELP-DCVAC therapy combined with ICIs (nivolumab alone or nivolumab and ipilimumab) until July 29. The outcome of this treatment against advanced HNC was diagnosed on August 22, 2022. By the endoscopy and CECT scan, it was diagnosed that all the cancers including the primary cancer mass and three LN-metastatic cancers (Figure 2D and Figures 3C, 3E, 3G) disappeared, although there was one swelling LN on the opposite left side of the neck. This enlarged LN was not detected at the next diagnosis; namely, this was noncancerous LN swelling, and it was also confirmed that all the cancers including primary cancer and three LN-metastatic cancers on the right side of the neck and left-sided neck swelling completely disappeared by a CT scan diagnosis on February 27, 2023. During the low-dose iCCI treatment, there were no serious side effects but a slight suspicion of self-healing interstitial pneumonia. The patient’s most recent CECT scan diagnosis on October 16, 2023, revealed no recurrence, and he has been cancer-free for over 18 months. The most recent diagnosis with a fiber endoscope on April 1, 2024, revealed that primary cancer has been eliminated for almost two years by iCCI treatment.

## Discussion

HNC is the sixth most common cancer worldwide and has a poor prognosis generally [1-4]. Therefore, it is important to explore optimized radiotherapy combined with other therapeutic modalities, which are superior for maintaining the patient’s quality of life (QOL), reducing adverse effects, and inducing a complete cure for cancer. To achieve a more powerful cancer treatment, most investigators have been focusing on evaluating the mechanisms of how a tumor-bearing host acquires antitumor immunity and how tumor-bearing hosts suffer from strong immunosuppression in TME. However, recent findings indicate the early activation of Th1 immunity in TDLN rather than TME is crucial for inducing fully activated CTL, which are final effector cells to destroy solid tumors [5-8,11]. Therefore, we developed iCCI consisting of radiotherapy, low-dose ICI therapy, and H/K-HELP-DC_VAC_, all of which induce an antitumor immunomodulating effect in TDLNs [5-8].

It is surprising that an inoperable LA-HNC was completely cured by iCCI without standard chemotherapy. Both 2 cm primary HNC and three LN metastatic cancers were completely removed by iCCI. There has been no recurrence of cancer, and the patient has still been cancer-free for almost two years by the most recent fiber endoscope diagnosis on April 1, 2024. Although the details of the cancer destruction mechanism remain unclear, we speculate that radiotherapy might induce the reprogramming of TDLNs and TME from “cold” immunosuppressive to “hot” immunostimulatory state, which is preferable to activate DC1 to recognize common cancer antigens (WT-1, survivin) [13,15] presented by DC and neoantigens and other antigens released from cancer cells by irradiation-induced immunological cell death (ICD) [16]. In a Th1-dominant state induced by ICIs, the generation of cancer-specific Th1 and CTL might be upregulated, and, conversely, immunosuppressive Treg is downregulated by Th1-derived IFN-γ [10]. Our theoretical speculation concerning tumor-rejection mechanisms of iCCI is strongly supported by others’ reports [11,16]. De Felice et al. [16] emphasized turning up to “hot” Th1-high condition from “cold” TME in a combination of radioimmunotherapy with ICIs. An indispensable role of neoantigen-specific IFN-γ-producing Th1 cells was elucidated for rejecting tumors by neoantigen-specific CTL even in cancer therapy models with neoantigen-vaccine and ICIs [11].

The antitumor efficacy of ICIs is derived from not only the release of strong immunosuppression but also the introduction of antitumor T cell responses in TDLNs and TME [6,12,17-19]. Indeed, CTLA-4/CD80, CD86 interaction strongly blocks SH-1/SH-2-mediated Th1 cytokine production, and it is canceled by anti-CTLA-4 blockade. Administration of CTLA-4 blockade, ipilimumab induces higher frequencies of ICOS^+^Th1-like CD4^+^ T cells rather than PD1 blockade [12,17]. Therefore, anti-CTLA-4 ipilimumab appears to be an even better inducer of Th1 immunity than anti-PD-1 nivolumab. However, the PD-L1-ligated PD-1/SHP-2 signaling pathway is also involved in Th1 cytokine production and nivolumab can restore robust Th1-dependent antitumor immunity in cancer patients [18].

Therefore, combination therapy with radiotherapy and ICIs is becoming a part of the standard treatment of intractable cancers including LA-HNSCC. However, only a subset of cancer patients benefit from this combination therapy. This might be because of insufficient cancer antigens released from cancer by radiotherapy-induced ICD or a weak activation of Th1 immunity in cancer patients. To overcome this problem, we further incorporated H/K-HELP-DC_VAC_ into iCCI.

H/K-HELP is an artificially synthesized long peptide (30-40 mer amino acids) cancer vaccine containing both CTL and Th1 epitopes [8,13,20]. In contrast to the classical short peptide vaccine, H/K-HELP acts as the elevated therapeutic vaccine to induce Th1 and CTL activation via efficiently sustained antigen presentation by professional DC at TDLNs in animal therapeutic models [8]. Moreover, it was indicated that H/K-HELP was an efficient peptide vaccine that could induce Th1-dependent cellular and humoral immune responses in clinical trials of human cancer [13,20]. We also confirmed that H/K-HELP-processed mature DC showed prolonged antigen-presenting capability compared with short peptide-pulsed DC in the human system [13]. Thus, H/K-HELP-DC_VAC_ might be suitable for supplementing a weak antigen release by radiotherapy alone and suppressing Th1-dependent antitumor immunity in cooperation with ICIs treatment, which facilitates the generation of CTL specific to common cancer antigens (WT-1 and survivin) expressing on most cancers. These CTLs coupled with radiotherapy-induced neoantigen-specific CTL might attack to immunotherapy-refractory hypopharyngeal SCC.

Thus, our designed iCCI combined with radiotherapy, low-dose ICIs-therapy, and H/K-HELP-DC_VAC_ will be a strong cancer therapeutic treatment without severe immune-related adverse effects of ICIs [14]. We believe that this new treatment (iCCI) will be effective in other patients with nasopharyngeal squamous cell carcinoma. Although the details of the destruction mechanism underlying iCCI are not determined, we speculate iCCI might improve a patient’s Th1-dependent antitumor immune responses in the immune microenvironment around tumors (IMAT) including TDLNs and TME. If cancer-specific immunological memory T cells were demonstrated in the cured patient, our developed iCCI would become a promising strategy to overcome inoperable advanced cancers in the future.

## Conclusions

We designed an iCCI comprising radiotherapy, ICIs, and H/K-HELP-DCVAC, all of which are known to modulate antitumor immunity in TDLNs to inhibit tumor growth. Surprisingly, the patient who suffered from stage IVA inoperable hypopharyngeal squamous cell cancer with 2 cm primary and three LN metastatic cancers was completely cured of cancer by treatment with our developed iCCI without standard chemotherapy, and he remained cancer-free for almost two years.
